# Femur tibial rotation in patients undergoing bilateral versus unilateral ACL reconstruction: A propensity score matched analysis

**DOI:** 10.1002/jeo2.70496

**Published:** 2025-10-30

**Authors:** Luca Farinelli, Fabrizio Di Maria, Amit Meena, Riccardo D'Ambrosi, Elisabeth Abermann, Christian Hoser, Christian Fink

**Affiliations:** ^1^ Department of Clinical and Molecular Sciences Clinical Orthopaedics Università Politecnica delle Marche Ancona Italy; ^2^ IRCSS INRCA Ancona Italy; ^3^ Department of General Surgery and Medical Surgical Specialities Section of Orthopaedics and Traumatology Università di Catania Catania Italy; ^4^ Gelenkpunkt ‐ Sports and Joint Surgery Innsbruck Austria; ^5^ Research Unit for Orthopaedic Sports Medicine and Injury Prevention (OSMI), Private University for Health Sciences, Medical Informatics and Technology Innsbruck Austria; ^6^ IRCCS Istituto Ortopedico Galeazzi Milan Italy; ^7^ Università Degli Dtudi di Milano Milan Italy

**Keywords:** anterior cruciate ligament, arthroscopy, knee, tibial rotation

## Abstract

**Purpose:**

The aim of the present study was to compare the tibial rotation between patients who have undergone unilateral versus bilateral ACL reconstruction (ACLR).

**Methods:**

Patients who underwent primary bilateral ACLR at our institution from the years 2010 to 2022 were retrospectively identified (*n* = 49). Exclusion criteria were: simultaneous injury (*n* = 2), not available magnetic resonance imaging (MRI) or surgical report (*n* = 13), or were older than 50 at the time of first ACL injury (*n* = 11). Twenty‐three patients were eligible for matching. A retrospective database analysis of consecutive unilateral primary ACL from 2019 to 2021 (*n* = 572) was performed. Patients with previous knee surgery, lateral collateral ligament and/or posterior cruciate ligament and/or posterolateral corner injury and fractures around the knee were excluded from the study. Those who had undergone unilateral ACLR with < 3‐year follow‐up were further excluded. Twenty‐three patients of case were matched with 96 unilateral ACL control by age, gender, body mass index and Tegner activity level. Two orthopaedic knee surgeons who were blinded measured the femur tibial rotation by measuring surgical epicondylar axes—posterior tibial condyles angle (SEA‐PTC angle) on MRI scans.

**Results:**

Propensity score matching analysis yielded 19 pairs of bilateral and unilateral ACLR. The mean SEA‐PTC angle in the bilateral ACLR group was −8.6° ± 3.9°. Specifically, the SEA‐PTC angle was −8.5° ± 3.4° and −8.7° ± 4.4° for right and left knee respectively. The mean SEA‐PTC angle in unilateral ACLR group was −5.2° ± 5.8°. The SEA‐PTC differed statistically between groups (*p* = 0.043).

**Conclusions:**

Patients who underwent bilateral ACLR had a significantly greater internal tibial rotation on MRI compared with those who underwent unilateral ACLR. Specifically, the mean of SEA‐PTC of bilateral group was about 1.5 times greater among those of unilateral ACLR.

**Level of Evidence:**

Level III, case–control study.

**Abbreviations**
ACL: anterior cruciate ligament
BMI: body mass index
ICC: inter‐rater correlation coefficient
LET: lateral extraarticular tenodesis
MRI: magnetic resonance imaging
SEA‐PTC: surgical epicondylar axes‐posterior tibial condyles
SMD: standardised mean difference

## INTRODUCTION

Anterior cruciate ligament reconstruction (ACLR) is one of the most common surgeries performed in orthopaedic sports medicine [4]. Anterior cruciate ligament (ACL) plays a central role in knee joint limiting anterior translation and internal rotation of the tibia relative to the femur [3, 15]. Vassalou et al. [19] indicated that adult knees demonstrated a mean 7° increase in the internal rotation when the ACL was ruptured by magnetic resonance imaging (MRI) analysis. Whereas, Mitchell et al. [16] found on MRI a significant increase in internal tibial rotation in ACL‐deficient knees compared to intact knees in the adolescent population. Hong et al. reported that aged patients with ACL tears exhibited significantly greater tibial internal rotation compared to younger patients (5.6° vs. 4.2°) hypothesising that older patients might have a higher incidence of associated injuries [12]. Indeed, it has been recently demonstrated that a greater internal tibial rotation in ACL injury patients has been associated to anterolateral ligament injuries, distal Kaplan fibre tears and medial meniscal ramp injuries [7, 8, 9]. Due to the retrospective design of previous studies [8, 9], cause‐effect relationship between greater internal tibial rotation and associated injuries could not be demonstrated, however, increased internal tibial rotation might represent an independent risk factor for rotatory knee instability in ACL injury setting with subsequent higher risk of associated lesions.

The aim of the present study was to compare the tibial rotation between patients who have undergone unilateral versus bilateral ACLR. It was hypothesised that patients who had a history of bilateral ACLR would have a significantly greater internal tibial rotation on imaging compared with patients who had a unilateral ACL tear.

## METHODS

### Study design

In this study, all the procedures involving human participants were performed in compliance with the 1964 Helsinki Declaration and its later amendments. The study protocol was approved by our institutional review board (AN2015‐0050 346/4.28).

The study was conducted according to the Strengthening the Reporting of Observational Studies in Epidemiology (STROBE) checklist. Informed consent was obtained from all the participants A retrospective analysis of prospectively collected data from the database of a specialised joint surgery clinic was conducted from 2010 to 2022. Two groups were constituted: primary non‐simultaneous bilateral ACLR (case) and primary unilateral ACLR (control).

As regard the first group, a retrospective analysis of all primary ACLR was performed from 2010 to 2022 in order to detect patients who had undergone bilateral non‐simultaneous ACLR. Patients excluded were those with: simultaneous ACL, over 50 age range at the time of first ACL injury, patients without magnetic resonance imaging (MRI) and/or surgical report available.

Regarding control group, consecutive primary ACLR performed from 2019 to 2021 (*n* = 572) were assessed for eligibility. Exclusion criteria: previous ipsilateral knee surgery, fractures around the knee, osteotomy, posterior cruciate ligament injury, lateral collateral ligament and/or posterior lateral corner injury, incomplete clinical data, MRI not available at out PACS. Concomitant meniscus treatment or lateral extra‐articular tenodesis (LET) were not considered as exclusion criteria.

Patients with unilateral ACLR were further excluded if they did not have a minimum of 3 years of clinical follow‐up. The reasoning for applying a 3‐year minimum threshold for the inclusion of patients with unilateral ACLR is that it could be hypothesised that patients with a shorter follow‐up after primary ACLR may potentially be predisposed to a subsequent contralateral ACL injury, but the injury may not yet have occurred.

**Figure 1:**
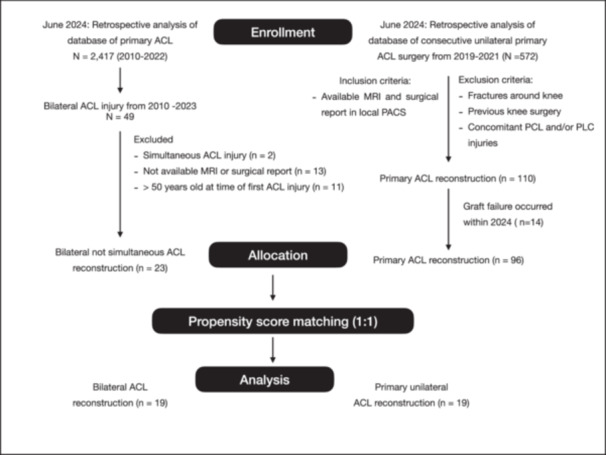
Flow chart of the study. ACL, anterior cruciate ligament; MRI, magnetic resonance imaging; PCL, posterior cruciate ligament; PLC, posterolateral corner.

Patients with bilateral ACLR were propensity score matched in a ratio of 1:1 on age, sex, Tegner activity level and body mass index (BMI), using the greedy nearest neighbour method, to patients with unilateral ACLR. A flow diagram for the inclusion of analysed patients is demonstrated in Figure 1.

### Internal rotation measurement

**Figure 2:**
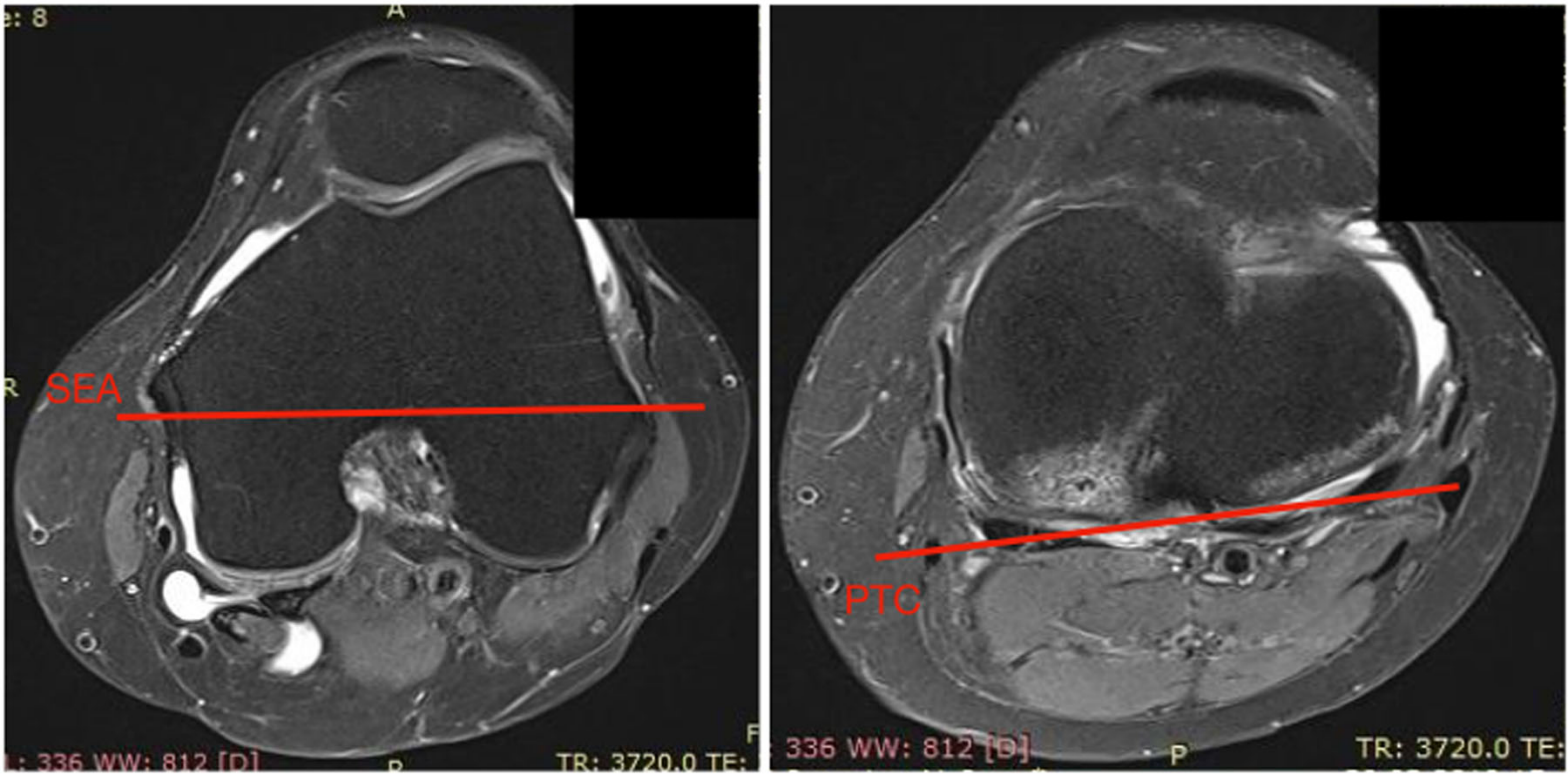
SEA‐PTC angle. PTC, posterior tibial condyles; SEA, surgical epicondylar axes.

To measure the axial alignment of the distal femur and proximal tibia, two sections were identified from each pre‐operative MRI [5]. The first slice was taken in the mid‐trochlear region of the femoral condyle, identified by the Roman arch appearance of the intercondylar groove with the apex of the Roman arch corresponding to one‐third of the height of the condyle. The surgical epicondylar axes (SEAs) from the lateral epicondyle and medial sulcus were delineated. The second slice was taken in correspondence with the proximal tibial plateau above the end of the proximal tibiofibular joint where the semimembranosus tendon inserts into the tibial bone. The tangent line of the posterior tibial condyles (PTCs) was delineated. The angle between SEA‐PTC was measured (Figure 2) [5, 6, 9]. A negative value was defined as internal torsion and a positive value as external torsion of the distal segment. All measurements were performed independently by two fellowship‐trained orthopaedic knee surgeons (L.F. and AM.) who were blinded to the patients' clinical history.

The SEA‐PTC angle was measured for each patient in unilateral group, whereas in bilateral group, the SEA‐PTC angle was measured for each side.

### Statistical analysis

A priori power analysis was performed to determine the appropriate sample size for the study. Considering an α level with *p* = 0.05, a power of 80%, and an effect size of 0.5, it was estimated that 17 matched pairs of participants would be needed respectively in 'case' and 'control' groups to detect a statistically significant difference in SEA‐PTC angle. The sample size calculation was performed with the use of the G‐Power software (G‐Power version 3.1) [14]. Data was collected and organised using Excel (Microsoft). Statistical analyses were performed using the XLSTAT statistical software packages (Addinsoft LLC). Categorical variables were expressed in numbers and percentages. The normal distribution of variables was verified through the Shapiro‐Wilk test. The angle SEA‐PTC did not show a normal distribution (*p* < 0.05). Mean and standard deviation or median and interquartile range (IQR) were used to summarise the variables according to their distribution. Differences in angles between the groups were assessed through the Wilcoxon rank‐sum test. Fisher exact test was used to assess significance between categorical variables. Inter‐rater correlation coefficient (ICC) was calculated for inter‐rater reliability for measuring SEA‐PTC. The significance threshold was set at *p* = 0.05. A post hoc power analysis was performed. We assessed the distribution of SEA‐PTC angle in 'case' and 'control'. A 10° of SEA‐PTC has been chosen as threshold for this study, therefore four groups (< −10°, −10° − 0°, 0° − 10°, > 10°) have been analysed. While there is no current consensus about the normal value of SEA‐PTC in ACL injured knee, Farinelli et al. previously found a cutoff of −10° of internal tibial rotation to be associated with 43% sensitivity and 96% specificity to identify patients with unstable medial meniscus ramp lesion in ACL injured knee [9].

### Propensity score matching

Patients of bilateral and unilateral were matched one‐to‐one by an optimal matching algorithm (Mahalanobis' distance). The algorithm identifies matched samples with the smallest average absolute distance across all matched pairs. This technique, regarded as an optimal method to evaluate differences between treatment groups, was applied to mitigate the impact of potential confounding variables. Patients were considered suitable for matching if the propensity score discrepancy between the groups fell within the caliper radius of 0.01×sigma [18]. The strength of the association and 95% confidence intervals were determined. The variables on which the two groups were harmonised included age, gender BMI and Tegner activity scale score. For bilateral group, the age of first time of ACL surgery for propensity score was considered. Discrepancies between the two groups were assessed by comparing the standardised mean difference (SMD) after matching. The matching was defined imbalanced for a particular covariate if the SMD exceeded 0.2 [2].

## RESULTS

### Study population and propensity score matching

The study flowchart with inclusion and exclusion criteria was shown in Figure 1. After a retrospective review of a total of 2417 patients from 2010 to 2022, 49 patients underwent bilateral ACL reconstruction. After exclusion criteria was applied, 23 patients (case) were available for propensity score matching. As regard control group, a total of 572 consecutive unilateral ACL reconstruction with a minimum of 3 years' follow‐up were identified from 2019 to 2021. After exclusion criteria were applied, there were 110 primary unilateral ACL reconstruction assessed for eligibility. Subsequently, 14 patients were further excluded due to graft failure with revision surgery occurred within June 2024. Finally, 96 patients were assessed for matching. Patients were matched 1:1 for a final cohort (*n* = 38) consisting of 19 in the bilateral group and 19 in the unilateral group with a total of 57 images (38 MRI for each patient of bilateral group and 19 MRI for unilateral group). Demographic data for the 38 included patients were reported in Table 1. The two matched groups showed a SMD of 0.14, 0.01 and −0.27 respectively of age, BMI and Tegner Activity level score. Unilateral group had a mean clinical follow up time of 3.65 ± 0.59 (range: 3.0–5.4) years. The mean time elapsed between ACLR in bilateral group was 4.05 ± 2.95 (range: 0.89–10.98). The post hoc analysis showed a power of 89% (G‐Power software v3.1) to find significant differences of SEA‐PTC angle between groups. The ICC value for the reliability of SEA‐PTC was 0.90, indicating an excellent agreement. Surgery data was reported in Table 2. The two groups were comparable in terms of graft, meniscus, cartilage and sMCL injuries. Lateral extraarticular tenodesis (LET) has been added in 5 (26%) patients of unilateral group compared to 0 and 1 (5%) of bilateral ACL (*p* = 0.039). Bilateral ACL reported a greater internal tibia rotation compared to unilateral matched control (*p* = 0.043). Hence, bilateral ACL reported an internal tibia rotation of −8.5° (3.4) and −8.7° (4.4) compared to −5.2 (5.8) of unilateral ACL. The overall mean of SEA‐PTC angle for bilateral group was −8.6 (3.9).

**Table 1:** Characteristics of the patients.

	Bilateral (*n* = 19)	Unilateral (*n* = 19)	*p* value
Male sex, *n* (%)	11 (57.9%)	13 (68.4%)	0.737
Age, mean (SD) [range]^a^	24.1 (10.1) [13–49]	25.4 (8.2) [15–52]	0.152
Body mass index, mean (SD) [range]^a^	21.7 (2.2) [18–26]	21.7 (1.7) [19–24]	0.925
Tegner activity level, mean (SD) [range]^a^	6.5 (2.1) [2–9]	7.0 (1.5) [4–10]	0.529

Abbreviations: ACL, anterior cruciate ligament; SD, standard deviation.

a Criteria of consideration was the age, body mass index and Tegner activity level at time of first ACL reconstruction for bilateral group.

**Table 2:** Surgery data and internal tibial rotation measurement.

	Bilateral (*n* = 19)		Unilateral (*n* = 19)	*p* value
Graft, *n* (%)	Right knee	Left knee		
Hamstring	8 (42%)	3 (16%)	2 (11%)	0.118
Quadriceps tendon	9 (47%)	14 (74%)	16 (84%)	
Patellar tendon	2 (11%)	2 (11%)	1 (5%)	
Lateral extraarticular tenodesis	0	1 (5%)	5 (26%)	**0.039**
Medial meniscus tear	6 (32%)	7 (37%)	12 (63%)	0.127
Lateral meniscus tear	8 (42%)	6 (32%)	10 (53%)	0.476
Both meniscus injuries	2 (11%)	4 (21%)	6 (32%)	0.341
Cartilage injuries	2 (11%)	3 (16%)	3 (16%)	1.00
sMCL injuries	7 (37%)	5 (26%)	7 (37%)	0.825
SEA‐PTC angle				
Mean, (SD)	−8.5 (3.4)	−8.7 (4.4)	−5.2 (5.8)	**0.043**
range	[−16, −3]	[−17, −1]	[−19, 1]	
Median, (IQR, 1–3 quartile)	−8 (4.5, −10.5 – (−6))	−8 (7, −12.5 – (−5.5))	−3 (8.2, −8.7 – (−0.5))	

*Note*: Bold values indicate statistically significant at p < 0.05.

Abbreviations: IQR, interquartile range; MCL, medial collateral ligament; SD, standard deviation.

**Figure 3:**
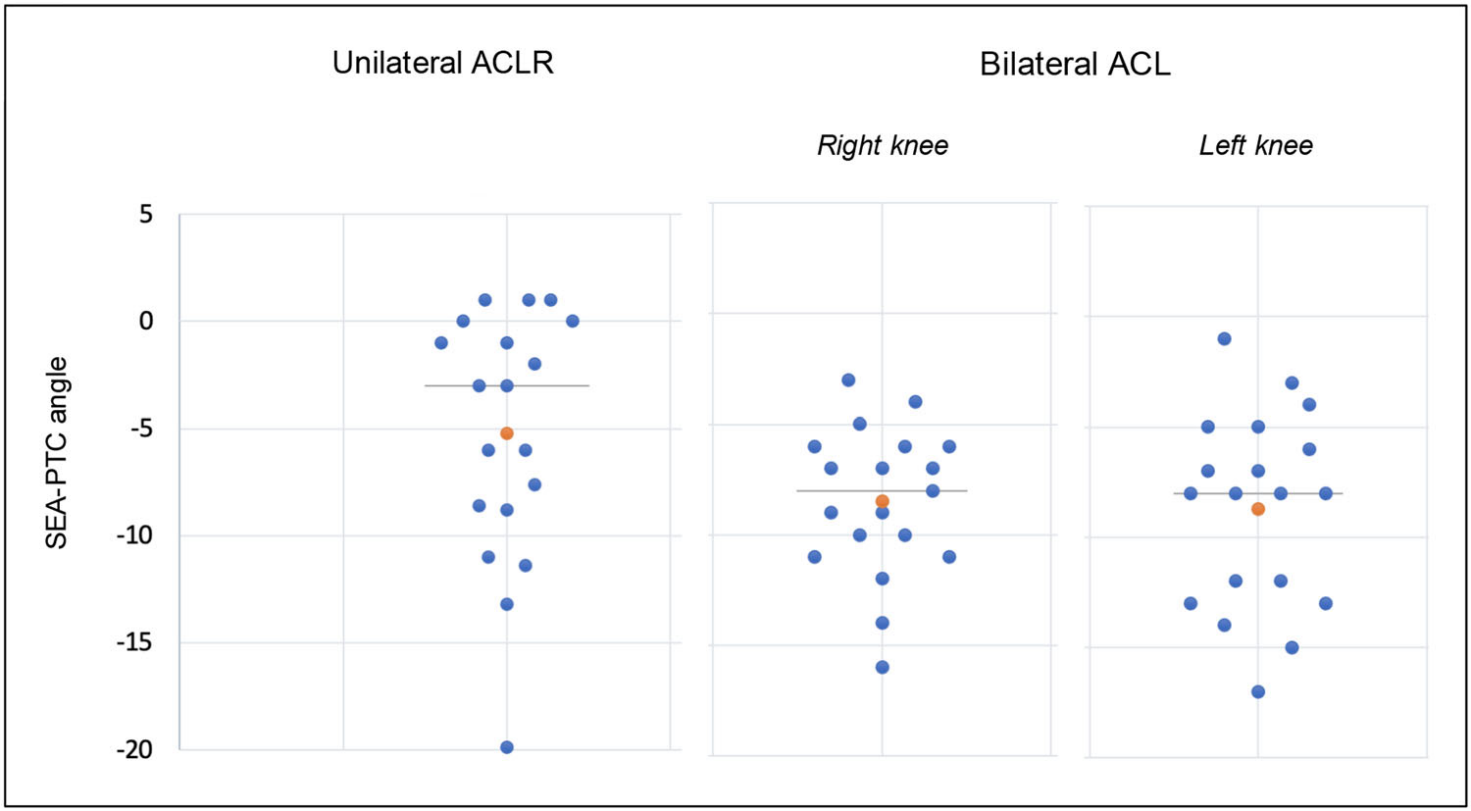
Distribution of SEA‐PTC angle of patients with unilateral and bilateral ACL. Red: mean, black line: median. ACL, anterior cruciate ligament; PTC, posterior tibial condyles; SEA, surgical epicondylar axes.

**Figure 4:**
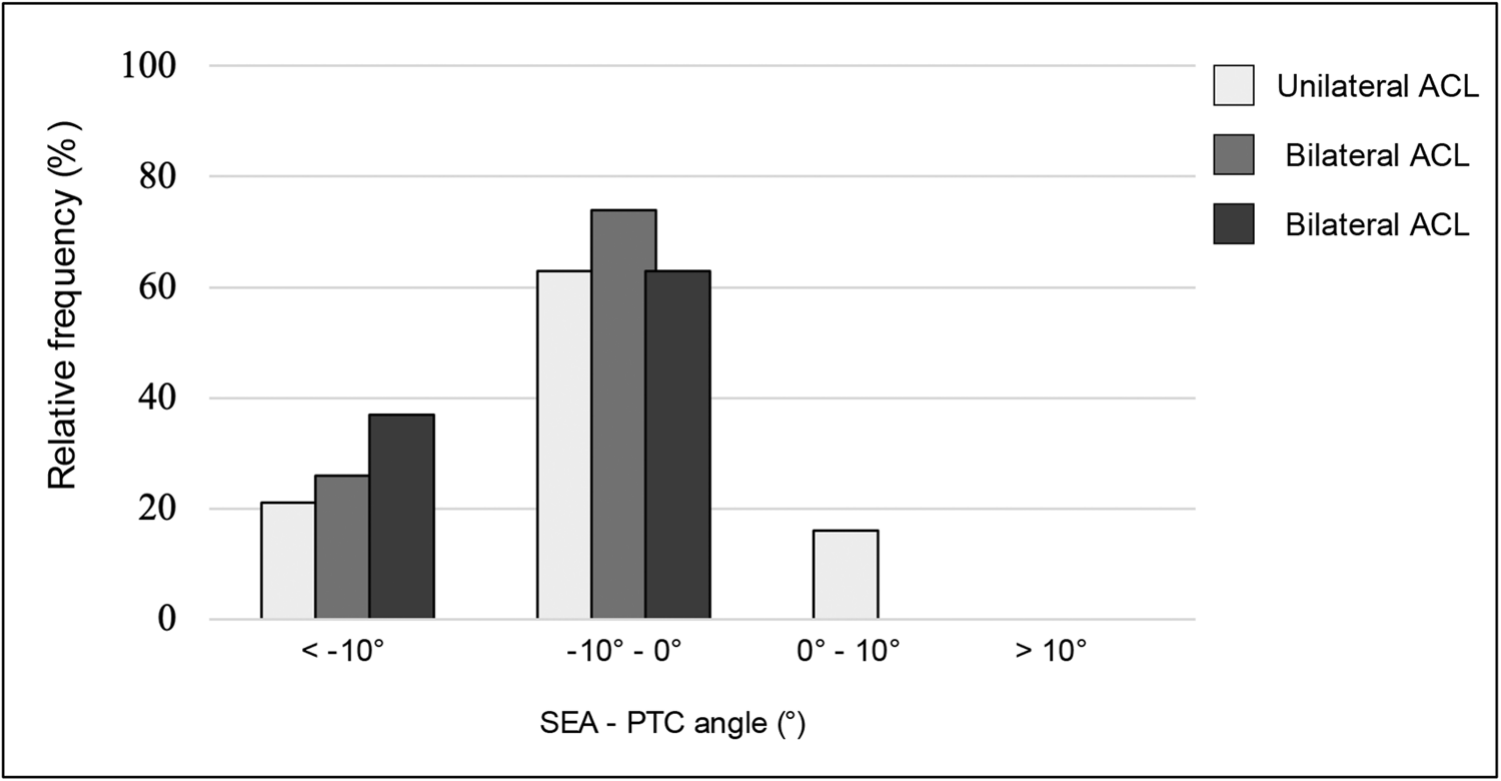
Comparison of SEA‐PTC angle distribution between the bilateral and unilateral groups. ACL, anterior cruciate ligament; PTC, posterior tibial condyles; SEA, surgical epicondylar axes.

The prevalence of SEA‐PTC measurements < 10° was 5 (26%) and 7 (37%) in the bilateral group, which were not significantly greater than the 21% (4 patients) prevalence in the unilateral group (*p* = 0.662). Twelve patients (63%) of unilateral ACLR were characterised by SEA‐PTC angle between −10° and 0°. The prevalence was similar in bilateral group with 12 (63%) and 14 (74%). Prevalence did not differ statistically between groups (*p* = 0.089). Three (16%) of patients of unilateral group were characterised by SEA‐PTC angle between 1° and 10°. No cases have been reported in bilateral group. The difference was not significant (*p* = 0.099). No patients of each group were characterised by an angle of SEA‐PTC angle greater than 10°. The distribution of SEA‐PTC angle of both groups were represented in Figures 3 and 4.

## DISCUSSION

The most important result of the present study was that patients who had undergone bilateral ACLR had a significantly greater tibial internal rotation than patients who had undergone unilateral ACLR. The association between internal tibial rotation and primary ACL injuries has been reported by a number of studies that measured the tibial rotation on MRI, comparing patients who sustained ACL injuries with those without a history of ACL injuries [12, 16, 19]. Moreover, previous studies found that patients with ACL tears and associated lesions exhibited significantly greater tibial internal rotation compared to controls [8, 9]. Similarly, Hong et al. found that adult patients with ACL tears exhibited significantly greater tibial internal rotation compared to adolescent patients, hypothesising that possibly associated injuries with ACL tears may increase tibial internal rotation in adolescent [12]. The detailed analysis of patients with bilateral non‐simultaneous ACL injury have been considered a valuable instrument to detect characteristics that may predispose a person to ACL injury [11]. In 1987 Houserworth et al. and Anderson et al. [1, 13] concluded that a narrowed posterior notch may predispose to ACL injury. More recently, Garra et al. [10] reported that patients who underwent bilateral ACLR had a significantly greater posterior tibial slope compared with those who underwent unilateral ACLR. From our results, patients who underwent bilateral ACLR had a significantly greater internal tibial rotation compared to those who underwent unilateral ACLR (Table 2) suggesting that femur‐tibia rotational alignment may have an important relationship with risk of ACL injury.

The results of the present study could be very useful in clinical daily practice. A greater internal tibia rotation could represent a novel risk factor for ACL injury as previous studies have reported for tibial slope [10]. Additional studies are required to establish the cutoff point values for diagnosis of ACL tears using femoral tibial rotation. Moreover, further analysis is required to assess if greater internal tibial rotation with ACL injury could represent a risk factor for graft failure.

The present study had several limitations that warrant disclosures. Due to its retrospective design, the study is inherently limited by selection bias. However, we attempted to reduce this bias through random selection from a large cohort of unilateral ACLR procedures as well as propensity score matching a comparison group based on age, sex, BMI and Tegner activity level score. The two groups showed an adequate balance with SMD of 0.14 and 0.01 respectively for age and BMI. Case and controls showed slight imbalance for Tegner activity with SMD of −0.27. Only patients with ACL complete tears were enroled in the present study therefore femoral tibia rotation from health controls were not included. The sample size of the matched cohorts was limited; however, the study was adequately powered (post hoc analysis showed a power of 89%). The present study has several strengths. Data from the current study were collected from a single medical centre. Effects of gender, BMI and Tegner activity level were avoided with propensity score matching. It has been reported that prevalence of LET was significantly higher in unilateral ACLR compared to bilateral group (26% vs. 0% and 5%, *p* = 0.039). A possible explanation was that patients in unilateral group had surgery in the last 5 years, time where most of studies about LET have been published [17]. Third, two orthopaedic surgeons measured SEA‐PTC angle on MRI, and the ICC of their measurements showed excellent agreement. Therefore, the measured data and analysed results in the present study were considered reliable.

## CONCLUSION

Patients who underwent bilateral ACLR had a significantly greater internal tibial rotation on MRI compared with those who underwent unilateral ACLR. Specifically, the mean of SEA‐PTC of bilateral group was about 1.5 times greater among those of unilateral ACLR.

## AUTHOR CONTRIBUTIONS

*Conceptualisation*: Luca Farinelli, Amit Meena, Christian Fink, Fabrizio Di Maria, and Riccardo D'Ambrosi. *Methodology*: Amit Meena, Luca Farinelli, Fabrizio Di Maria and Christian Hoser. *Data curation and synthesis*: Luca Farinelli, Amit Meena, and Riccardo D'Ambrosi. *Writing—original draft preparation*: Luca Farinelli, Amit Meena, and Elisabeth Abermann. *Writing—review and editing*: Amit Meena, Luca Farinelli, and Christian Fink. *Supervision*: Christian Hoser, Elisabeth Abermann, and Christian Fink. All authors interpreted the data, critically reviewed the work, made important contributions to the manuscript with their suggestions for improvement, approved the published version and agreed to be responsible for all aspects of the work. All authors have read and agreed to the published version of the manuscript.

## CONFLICT OF INTEREST STATEMENT

One of more authors has declared a potential conflict of interest as specified in the ICMJE conflict of interest statement.

## ETHICS STATEMENT

All procedures performed in studies involving human participants were in accordance with the ethical standards of the institutional and/or national research committee and with the 1964 Helsinki declaration and its later amendments or comparable ethical standards. The study protocol was approved by our institutional review board (AN2015‐0050 346/4.28). Informed consent was obtained from all individual participants included in the study.

## Data Availability

Available if required.
